# Lung Adenocarcinoma With Intratumoral Necrosis Induced by Bronchial Artery Embolization

**DOI:** 10.1002/rcr2.70520

**Published:** 2026-02-19

**Authors:** Ikumi Kashiwagi, Akihiko Sokai, Mamoru Takahashi, Akihiro Takahagi, Harutaro Okada, Toshiyuki Iwata, Yasuyuki Hayashi, Yuki Sakai, Naoaki Yasuda, Miki Tasato, Saki Kusunoki, Toshihide Yamaoka, Takashi Nishimura

**Affiliations:** ^1^ Department of Respiratory Medicine Kyoto Katsura Hospital Kyoto Japan; ^2^ Department of Thoracic Surgery Japanese Red Cross Otsu Hospital Otsu Shiga Japan; ^3^ Department of Thoracic Surgery Kyoto Katsura Hospital Kyoto Japan; ^4^ Department of Radiology Kyoto Katsura Hospital Kyoto Japan

**Keywords:** adenocarcinoma, bronchial artery embolization, cavitation, hemoptysis, lung cancer

## Abstract

A 70‐year‐old woman presented with hemoptysis due to a right upper lobe mass. Bronchial artery embolization (BAE) using gelatin sponge was successful in controlling bleeding. Two weeks later, chest computed tomography (CT) revealed that the solid tumour had been replaced by a 5.6 cm thin‐walled cavity without fluorodeoxyglucose uptake on positron emission tomography–computed tomography, although fluorodeoxyglucose accumulation was observed in the right hilar lymph node. Right upper lobectomy confirmed adenocarcinoma. The patient, confirmed with a pathological stage of IIIA, received adjuvant chemotherapy and has remained recurrence‐free for more than 5 years. This case suggests that BAE induced tumour necrosis and cavitation; however, viable tumour tissue remained. Further accumulation of similar cases is needed to clarify the therapeutic significance of BAE in lung cancer.

## Graphical abstract

We report a case of lung adenocarcinoma in which bronchial artery embolization induced tumour necrosis and cavitation, facilitating curative resection and leading to long‐term disease‐free survival.

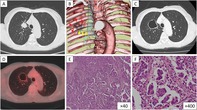

## 1 Introduction

Hemoptysis occurs in some patients with lung cancer and may be life‐threatening. Bronchial artery embolization (BAE) is a well‐established procedure for hemostatic control, while its effect on tumour structure and pathology remains unclear. We report a case of lung adenocarcinoma in which BAE induced tumour necrosis and cavitation, facilitating curative resection and leading to long‐term disease‐free survival.

## 2 Case Report

**FIGURE 1:**
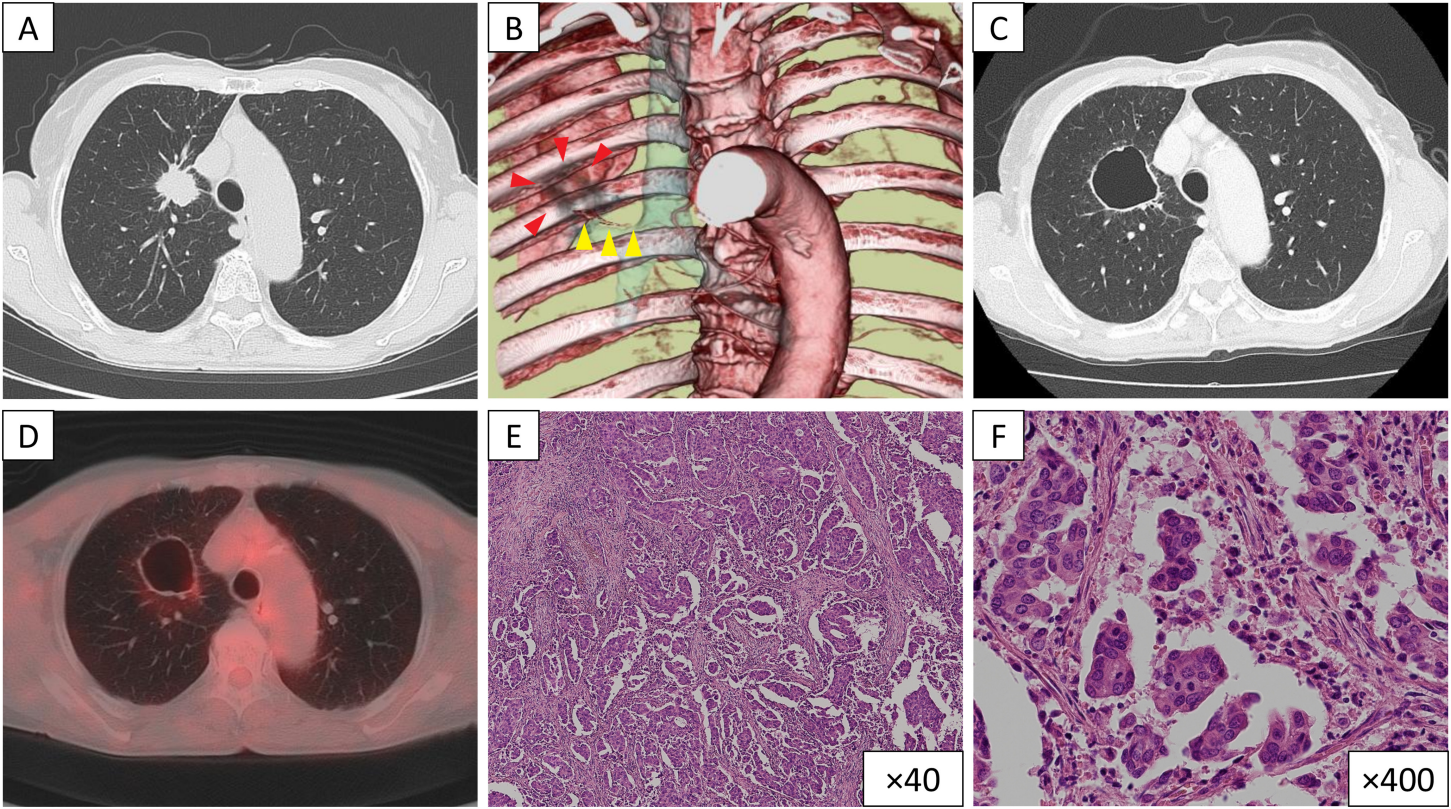
(A) Chest computed tomography (CT) at admission shows a 3 cm nodule in the right upper lobe. (B) Three‐dimensional vascular reconstruction image prior to bronchial artery embolization (BAE) shows a dilatation of the bronchial artery (yellow arrow heads) arising from the descending aorta, supplying the tumour (red arrow heads). (C) Chest CT after BAE shows replacement of the right upper lobe nodule by a thin‐walled cavitary lesion with a maximum diameter of 5.6 cm. (D) Positron emission tomography–computed tomography image after BAE shows no fluorodeoxyglucose uptake at the cavitary lesion. (E, F) Pathological findings of the right upper lobectomy specimen show extensive adenocarcinoma. Haematoxylin and eosin staining, ×40 (E) and ×400 (F).

A 70‐year‐old woman was urgently admitted to our hospital for a two‐day history of hemoptysis. She was a current smoker and had no medical history other than an Achilles tendon rupture. The physical examination revealed blood pressure of 142/86 mmHg, an oxygen saturation of 98% on room air, and a respiratory rate of 16 breaths/min. Laboratory findings showed haemoglobin of 12.2 g/dL and C‐reactive protein of 0.02 mg/dL. Chest computed tomography (CT) showed a mass with a maximum diameter of 3 cm in the right upper lobe supplied by two bronchial arteries from the descending aorta (Figure 1A,B). Retained bloody secretions were observed in the distal bronchi, and ground‐glass opacity suggested hemorrhagic aspiration. BAE was performed using a gelatin sponge sheet (Serescue; Astellas, Tokyo, Japan) and resolved the hemoptysis.

Chest CT revealed a cavitary lesion with a thin wall and a maximum diameter of 5.6 cm in the right upper lobe instead of the solid tumour, 2 weeks after BAE (Figure 1C). On positron emission tomography–computed tomography images, the cavitary lesion showed no fluorodeoxyglucose uptake (Figure 1D), but fluorodeoxyglucose accumulation was observed in a single mass consisting of the right hilar lymph node and the tumour. The patients underwent right upper lobectomy after bronchoscopic diagnosis of adenocarcinoma. Pathological findings of the surgical specimen revealed adenocarcinoma with mediastinal hilar lymph nodes metastasis and the primary tumour, and T1cN2M0 corresponding to pathological stage IIIA (Figure 1E,F). Molecular marker analysis was not performed because the amount of tumour tissue was insufficient for further testing. Thereafter, she was administered adjuvant chemotherapy with cisplatin plus S‐1, and radiotherapy was not performed. She has remained free of recurrence for more than 5 years.

## 3 Discussion

Patients with lung cancer occasionally develop hemoptysis, which can be life‐threatening in approximately 10% of these cases [1, 2]. Life‐threatening hemoptysis in lung cancer is most often caused by bronchial arterial hypervascularization, while pulmonary artery rupture accounts for only a minority of cases [2]. BAE is an established method for controlling hemoptysis caused by lung cancer [2]. While BAE for hemoptysis due to lung cancer is primarily intended for haemostasis, transarterial embolization (TAE) or transarterial chemoembolization (TACE) in hepatocellular carcinoma is widely recognised not only for its palliative efficacy but also for its ability to induce tumour necrosis and regression [3]. While BAE is not a definitive therapy in lung cancer, a case report showed that BAE led to massive tumour necrosis and successful subsequent surgical resection [4]. In the present case, post‐BAE enlarged cavitation may have resulted from ischemic necrosis combined with a check‐valve mechanism due to bronchial involvement. Similar mechanisms have been described after radiofrequency ablation, where tumour necrosis and bronchial encasement have been identified as factors contributing to early enlarging cavitation [5].

Although the present case demonstrated that BAE led to the formation of a thin‐walled cavity and enabled subsequent surgical resection, viable tumour cells remained within the cavity wall. Therefore, BAE should be regarded merely as a hemostatic measure for controlling hemoptysis, and surgical resection remains necessary as a definitive treatment for lung cancer. It seems difficult for BAE to serve as a curative therapeutic modality comparable to TAE or TACE in hepatocellular carcinoma. Further accumulation of similar cases and detailed evaluation is required to clarify whether BAE plays a supplementary role in surgical resection.

## Funding

The authors have nothing to report.

## Consent

The authors declare that written informed consent was obtained for the publication of this manuscript and accompanying images and attest that the form used to obtain consent from the patient complies with the Journal requirements as outlined in the author guidelines.

## Conflicts of Interest

The authors declare no conflicts of interest.

## Data Availability

The data that support the findings of this study are available on request from the corresponding author. The data are not publicly available due to privacy or ethical restrictions.
